# Epidemiology of 30,000 Pediatric Urgent Care Telemedicine Visits in the Era of COVID-19

**DOI:** 10.1089/tmj.2021.0482

**Published:** 2022-10-07

**Authors:** Mordechai D. Raskas, Gabriel J. Feuerstein-Mendik, Gary Gerlacher, Sheryl Cohen, Shannon Henning, Jennifer M. Cramer, Ora Sultan, Sabah F. Iqbal

**Affiliations:** ^1^ Division of Telemedicine, PM Pediatrics, Lake Success, New York, New York, USA.; ^2^ Department of Pediatrics, Georgetown University School of Medicine, Washington, District of Columbia, USA.; ^3^ Program in Public Health Studies, Krieger School of Arts and Sciences, Johns Hopkins University, Baltimore, Maryland, USA.

**Keywords:** COVID, telemedicine, telehealth, pediatrics, pandemic

## Abstract

**Background:**

To describe the epidemiology of patients accessing a pediatric urgent care telemedicine platform during the COVID-19 pandemic.

**Study Design:**

We conducted a cross-sectional study of the first 30,000 pediatric patients who accessed our pediatric urgent care telemedicine platform during the beginning of the COVID-19 pandemic. The study population came from 15 states and included the dates May 15 through September 16, 2020. We also described the groups of patients referred for in-person evaluation in urgent care or emergency department (ED) settings.

**Results:**

Mean patient age was 7.6 ± 5.4 years and 51% of patients were male. Twenty-one percent were publicly insured. More than 60% of patients sought care between 12 and 7 p.m. The most common reasons for seeking care were concerns for COVID-19 (50.5%) and fever (6.8%). Antibiotics were prescribed in 4.3% of visits. Children had an in-person visit to our urgent care offices on the same day in 9% of visits. Less than 1% of children were referred to the ED.

**Conclusions:**

In this large series of telemedicine visits during the COVID-19 pandemic, fewer than 10% required escalation to an in-person office visit and fewer than 1% required escalation to an ED.

## Background

The COVID-19 pandemic has had far reaching consequences on the functioning of the U.S. health care system. With hospitals and emergency departments (EDs) overwhelmed across the country, and rising SARS-CoV-2 morbidity and mortality, many patients became fearful of entering the health care system. In 2020, from March 29 to April 25, pediatric ED visits decreased by ∼70% for children aged 0–10 and by ∼50% for children aged 11–24.^1^ This underutilization, thought to be due to fear of contracting COVID-19 in a healthcare setting, resulted in an increase in delayed diagnoses of serious illnesses.^2–4^

With such a dramatic decrease in willingness to enter health care facilities, combined with overcrowding in many hospitals around the nation, it became critically important to provide urgent medical services remotely via telemedicine. Telemedicine can be defined as “the use of information technology to provide clinical health care from a distance.”^5,6^ Many different types of medical services have been provided in this manner, ranging from telepsychiatry to postoperative checks, with well-documented success.^7–9^

Several studies describe how to set up pediatric telemedicine systems both before and during the COVID-19 pandemic, yet few focus on acute care telemedicine and none describes the epidemiology and clinical characteristics of the patients who sought care via telemedicine during the pandemic.^10–13^ Data regarding the effectiveness of telemedicine for caring for children are lacking, as are data on how often children need further in-person evaluation after telemedicine appointment. While the COVID-19 pandemic will eventually (hopefully) subside, many families will continue to seek telemedicine care from home, at a time when most convenient for the family.

Since the start of the COVID-19 pandemic, PM Pediatrics, the nation's largest pediatric urgent care group, has conducted more than 150,000 pediatric urgent care telemedicine visits. Our telemedicine was in early stages before COVID-19, with fewer than 1% of our visits being telemedicine in February 2020 and then rapidly expanded due to COVID-19. Telemedicine visits peaked at 60% of total visits in May 2020, and then quickly declined in the months following to 10–20% of total visits.

Due to the fact that our in-person clinical operations remained functional during the entire pandemic, we are in a unique position to characterize the pediatric patients who seek telemedicine care in the setting of the COVID-19 pandemic. Given that telemedicine is known to have higher rates of antibiotic prescribing than in-office care, we also wanted to evaluate antibiotic prescribing rates on telemedicine for a pediatric urgent care population.

A study by Alpern et al. was the first to define the population of patients who present to pediatric EDs and gave foundational understanding to the epidemiology of pediatric emergency medicine; similarly, we hope that our study will define the epidemiology of acute pediatric telemedicine visits and, like the study by Alpern et al., will inspire additional research and knowledge in this nascent field.^14^ This study was approved by Solutions IRB.

## Methods

### TELEMEDICINE PLATFORM

Our HIPAA-compliant (Health Insurance Portability and Accountability Act-compliant) platform, PM Pediatrics Anywhere, is available through either a website or a mobile app. It is an on-demand model, where, after entering their child's name and demographic information, medical history, insurance information, and pharmacy, patients are immediately connected with the first available urgent care provider licensed in their state. There are no scheduled appointments. The platform is based on industry standard WebRTC protocols for encoding and decoding and delivers a real-time, high-quality, and interactive audio and video communication technology. Peripheral devices, such as otoscopes and stethoscopes, were generally not available to patients in their homes.

Patients met with one of the following four types of providers: board-certified pediatric emergency medicine physicians, board-certified pediatricians, nurse practitioners, or physician assistants. All our providers were also employed in our in-person operations, all of which follow evidence-based guidelines in the diagnosis and management of pediatric illness and injury. Operating procedures from the American Telemedicine Association were followed,^15^ and shortly after the study, in November 2020, our telemedicine program received accreditation by URAC, an independent, nonprofit accreditation entity.^16^

In this study, pediatricians responded to 17,111 (57%) visits, and pediatric emergency medicine physicians responded to 6,214 (20.7%) visits. Unlike prior studies,^17^ in our telemedicine platform, we do not exclude any patients based on chief complaints, and instead, evaluated all patients who presented to the platform. Providers obtained a standard history from the parent, guardian, and child, and then visually examined the patient, utilizing guardian assistance as needed, to assess for palpable findings. At the conclusion of the visit, a full copy of the visit record was made available to the parent.

### SELECTION OF PARTICIPANTS

All children aged 0–18 who presented for a telemedicine visit were included in this study. Patients were excluded if they were 19 or older or had an incomplete or unsigned electronic medical record. The PM Pediatrics telemedicine system covered the following 15 states: AK, CA, CT, FL, IL, MD, MA, NJ, NY, NC, PA, TN, TX, VA, and DC. We had urgent care offices in all but FL and DC, where site openings were delayed due to the pandemic. All offices were operational during the data collection period and gained COVID-19 testing capabilities. COVID-19 testing in our urgent care offices was available both via walk-in and after a telemedicine screening.

### TIMING OF STUDY

The PM Pediatrics telemedicine system was initially launched on March 9, 2020, in response to the COVID-19 pandemic, and in 2 weeks was implemented in all states in which we had a physical presence, as well as in FL and DC. By May 15, the telemedicine program was fully operational. Telemedicine was available from 8 through 12 a.m. every day; however, our offices also remained open for in-person care. In addition, we had COVID-19 testing capabilities in all regions. We conducted a retrospective chart review of the first 30,000 pediatric telemedicine visits, starting on May 15, 2020, and concluding on September 16, 2020.

### DATA COLLECTION

We obtained data from our electronic health record, eClinicalWorks (v. 2020; eClinicalWorks, Westborough, MA), and PM Pediatrics Anywhere, our telemedicine application (v. 2020; Teladoc Health, Inc., Purchase, NY). Data aggregation and analysis took place in our data system, Qlik Sense (v. 2020; Qlik, King of Prussia, PA), Excel (v. Office 365 2012; Microsoft Corporation, Redmond, WA), and RStudio (v. 2021; RStudio, PBC, Boston, MA). Data elements included age at visit, insurance, state, time of day of visit, preferred language, wait time, chart completion time, gender assigned at birth, chief complaint, and International Classification of Disease (ICD)-10 diagnosis code. We also describe patients who required immediate transfer to the ED with those referred to our urgent care offices for immediate care. For patients who were transferred to our pediatric urgent care offices, we recorded their current procedural terminology (CPT) codes and ICD-10 diagnosis codes from their in-person visits.

We also analyzed COVID-19 testing outcomes and reviewed patient satisfaction surveys.

In addition, we compared the basic demographic data of our study population with our prepandemic population of in-office patients who were seen in the 4 months before the pandemic.

### ANALYSIS

This study was descriptive, and all data were summarized with descriptive measures. Continuous variables were described using means and medians as appropriate and discrete variables were described using counts and proportions. Given the importance of the Alpern et al. study in defining pediatric emergent care, we modeled their methods to describe our population.^14^

## Results

### PATIENT POPULATION

Of our population of 30,000 visits, 51% were male and mean patient age was 7.25 years. This is minimally different from the population seen in office in the 4-month period before the pandemic, where mean age was 6 years (*p* < 0.05) and 50.3% were male (*p* = 0.03) (*Table 1*). The majority of telemedicine patients had private insurance (74.1%), while 21% had public insurance and 4.9% were uninsured. The telemedicine patients had a significantly higher percentage of private insurance than patients seen in the 4-month period before the pandemic (65.9%, *p* < 0.05). While the patient populations before and after the pandemic differed significantly in terms of age, percent male, and insurance status, it is unlikely that these differences are clinically significant.

**Table 1.** Demographic Data

VARIABLE	*N*	PERCENT OF TOTAL
Male patients	15,292	51
Age		
Infants (0–2)	4,391	14.6
Childhood (2–12)	17,576	58.6
Adolescent (12–19)	8,033	26.8
Insurance status		
Private	22,233	74.1
Public	6,284	21
Self-pay/uninsured	719	2.4
Unknown	764	2.5
Patient's home state (no. of offices)		
Alaska (1)	296	1
California (1)	689	2.3
Connecticut (1)	511	1.7
Florida (0)	190	0.6
Illinois (1)	72	0.2
Maryland (9)	4,169	13.9
Massachusetts (2)	819	2.7
Michigan (0)	1	0.003
New Jersey (10)	7,457	24.9
New York (19)	8,201	27.3
North Carolina (2)	2,328	7.8
Pennsylvania (4)	1,802	6.0
Tennessee (1)	15	0.05
Texas (4)	1,129	3.8
Virginia (4)	1,899	6.3
Washington, DC (0)	422	1.4
Preferred language other than English	298	0.01
Time of day of visit		
8:00–12:00	6,675	22.3
12:00–16:00	10,331	34.4
16:00–20:00	9,037	30.1
20:00–0:00	3,957	13.2
Patients with repeat visits	2,463	9.1
Visits referred for in-person COVID-19 testing	17,864	59.6
Wait time <15 min	25,437	84.8

Over 50% of our total visit volume was from patients located in NY (27.3%) and NJ (24.9%), where the majority of our physical offices are located and where we have had a physical presence for the longest time. Similarly, during the 4-month period before the pandemic, 44% of our total in-office visits were in NY and 22% were in NJ. The vast majority (95%) of patients were located within 20 miles of a PM Pediatrics office. We measured repeat visits on the telemedicine platform within our 4-month study period and found that nearly 10% of our total visits were repeat visits. While the telemedicine platform is presented in English, there are both American Sign Language and foreign language interpretation capabilities built into our system, however, only 0.01% of our population identified as non-English speaking or deaf patients seeking telemedicine care.

Telemedicine was available from 8 to 12 a.m.; 64.6% (19,368) of telemedicine visits occurred between 12 and 7 p.m. Median wait time to see a provider was 2.7 min and 84.8% of patients were seen within 15 min. The median time from start of the visit to chart completion for all providers was 16.3 min. A total of 59.5% (17,861) of the visits were screening visits for COVID-19 testing at our urgent care offices. *Table 1* shows demographic data for the study population.

### CHIEF COMPLAINT AND DIAGNOSES

We analyzed chief complaints in our study population (*Table 2*). Visits for screening for COVID-19 testing were the most common chief complaint, with 55.4% of patients requesting testing or seeking medical care for concern of COVID-19. Of those visits for COVID-19 testing, the mean age was slightly higher (9.3 ± 5.4 years) compared with the general telemedicine visit population (7.6 ± 5.4 years, *p* < 0.001). The next most common chief complaint was fever (6.8%), with a mean age of 4.1. When we excluded patients seeking screening for COVID-19 testing, fever (15.3%) and rash (14.6%) were the most common chief complaints for seeking urgent care via telemedicine. The remaining chief complaints included ear pain, bites and stings, red or irritated eye, skin and soft tissue infections, and upper respiratory complaints. Allergic reactions also made up a prominent portion (1.1%) of our visits.

**Table 2.** Most Frequent Chief Complaints

CHIEF COMPLAINT (30,000)	*N*	% OF TOTAL	% OF TOTAL EXCLUDING COVID-19	% MALE	MEAN AGE
COVID-19	16,633	55.4	—	50.5	9.3
Fever	2,044	6.8	15.3	52.5	4.1
Rash	1,950	6.5	14.6	53.6	4.3
Ear pain	1,083	3.6	8.1	49.2	7.3
Insect bite	822	2.7	6.1	49.8	4.8
Red eye	567	1.9	4.2	54.7	5.3
Skin infection	557	1.9	4.2	50.4	5.9
Sore throat	465	1.6	3.5	43.4	8.8
Cough	384	1.3	2.9	54.7	5.8
Allergy	338	1.1	2.5	57.1	4.7

Using ICD-10 diagnosis codes, we evaluated the most common diagnoses of the study population (*Table 3*). In our telemedicine software platform, each visit was reported with exactly one ICD-10 code. ICD-10 codes that were being used by our providers for COVID-19 screenings (Z11.59, Z20.828, Z20.89, Z03.818, and Z 20.9) were aggregated into one diagnosis category. The most common diagnosis was COVID-19/COVID-19 screening (52.1%). Fever was the second-most common diagnosis code used and rash was third.

**Table 3.** Most Frequent Diagnoses

DIAGNOSIS (30,000)	*N*	% OF TOTAL^a^	% OF TOTAL EXCLUDING COVID-19	% MALE	MEAN AGE
COVID-19/COVID-19 screening (see list below for ICD-10 codes)	15,633	52.1	—	51	7.6
Fever, unspecified [R50.9]	2,157	7.2	15	51.9	4.3
Rash and other nonspecific skin eruption [R21]	1,003	3.3	7	52.9	4.2
Acute upper respiratory infection, unspecified [J06.9]	610	2.0	4.3	46.9	5.7
Cough [R05]	421	1.4	2.9	52	6.5
Viral infection, unspecified [B34.9]	395	1.3	2.8	54.7	5.9
Acute pharyngitis, unspecified [J02.9]	366	1.2	2.6	44	9.3
Diarrhea, unspecified [R19.7]	213	0.7	1.5	54.5	4.7
Dysuria [R30.0]	184	0.6	1.3	17.9	5.5
Unspecified injury of head, initial encounter [S09.90XA]	154	0.5	1.1	57.8	2.8
Vomiting, unspecified [R11.10]	150	0.5	1.0	54.7	4.7

^a^ Denominator for percent is total number of telemedicine visits. Exactly one ICD-10 code reported per visit.

ICD, International Classification of Disease.

Note: COVID-19/COVID-19 Screening ICD-10 codes: Encounter for screening for other viral diseases [Z11.59]. Contact with and (suspected) exposure to other viral communicable diseases [Z20.828]. Contact with and (suspected) exposure to other communicable diseases [Z20.89]. Encounter for observation for suspected exposure to other biological agents ruled out [Z03.818]. Contact with and (suspected) exposure to unspecified communicable disease [Z20.9].

### ANTIBIOTIC PRESCRIPTIONS

We found that 6.9% of visits in May had an oral antibiotic prescribed, 4.2% in June, 3.6% in July, and finally, only 3.3% in August. Overall, oral antibiotics were prescribed in 1,282 (4.3%) visits. The most common diagnoses associated with oral antibiotic prescription were skin infections (35%), wounds (11.5%), impetigo (10.5%), and otitis media (8.7%).

### REFERRAL FOR IMMEDIATE CARE

After the telemedicine visit, we saw 2,266 patients in-person at one of our urgent care offices on the same day as their telemedicine visit. The most common ICD-10 codes associated with these visits were related to COVID-19 in 1,088 (Z11.59, Z20.828, Z20.89, Z03.818, and Z20.9), pharyngitis/tonsillitis in 519 (J02.9, J02.0, and J03.90), and fever in 391 (R50.9). The top 10 diagnosis groups along with the most frequent ICD-10 codes are shown in *Table 4*. We also evaluated the CPT codes associated with these visits and found that there were 656 (28%) CPT codes assigned to these 2,266 visits. In approximately one-fourth of visits, patients who presented for same-day care required one or more of the following: blood or urine testing (11.6%), radiography (5.9%), laceration repair (5.2%), splint/immobilization (4.3%), abscess drainage (0.8%), or foreign body removal (0.6%).

**Table 4.** Most Frequent Diagnosis Groups and International Classification of Disease-10s for Same-Day In-Person Urgent Care Visits

DIAGNOSIS GROUP AND MOST FREQUENT DIAGNOSES	ICD-10 CODE	VISIT COUNT
Factors influencing health status and contact with health services	—	1,137
Contact with and (suspected) exposure to other viral communicable diseases	Z20.828	790
Encounter for screening for other viral diseases	Z11.59	298
Symptoms, signs, and abnormal clinical and laboratory findings, not elsewhere classified	—	872
Fever, unspecified fever cause	R50.9	279
Dysuria	R30.0	168
Diseases of the respiratory system	—	724
Sore throat	J02.9	372
Viral upper respiratory tract infection	J06.9	58
Injury, poisoning, and certain other consequences of external causes	—	458
Facial laceration, initial encounter	S01.81XA	22
Chin laceration, initial encounter	S01.81XA	18
Diseases of the ear and mastoid process	—	227
Acute swimmer's ear of left side	H60.332	14
Acute suppurative otitis media of left ear without spontaneous rupture of tympanic membrane, recurrence not specified	H66.002	14
Infectious and parasitic diseases	—	215
Viral syndrome	B34.9	76
Viral illness	B34.9	42
Diseases of the skin and subcutaneous tissue	—	121
Abscess	L02.91	14
Eczema, unspecified type	L30.9	5
Diseases of the genitourinary system	—	108
Acute vaginitis	N76.0	25
Urinary tract infection	N39.0	12
Diseases of the digestive system	—	67
Gastroenteritis	K52.9	15
Constipation, unspecified constipation type	K59.00	13
External causes of morbidity and mortality	—	54
Bitten or stung by nonvenomous insect and other nonvenomous arthropods, initial encounter	W57.XXXA	15
Insect bite, unspecified site, initial encounter	W57.XXXA	9

ED referral occurred in 219 visits (0.7%). ED referrals were more likely in the late evening hours; nearly 40% of ED referrals occurred during the hours of 8 p.m. to 12 a.m. (*Table 5*), likely due to both telemedicine and PM Pediatrics offices closing, requiring pressing health concerns to be taken to an ED. Overall, the population of patients who were referred to an ED had similar sex, state, and insurance status distributions to the overall study population.

**Table 5.** Visits and Emergency Department Referrals by Hour of Day

HOUR OF DAY	VISITS	NO. OF VISITS RESULTING IN ED REFERRAL (%)
8–12	6,675	32 (14.6)
12–16	10,331	40 (18.3)
16–20	9,037	61 (27.9)
20–24	3,957	86 (39.3)
Total	30,000	219 (100)

ED, emergency department.

ED referral visits had a younger mean age (5.1 ± 5.1 years) compared with the general telemedicine visit population (7.6 ± 5.4 years, *p* < 0.001). Fever (*n* = 39) was the most common diagnosis resulting in ED referral (*Table 6*). Other common causes for referral included respiratory distress, vomiting, and urticaria, and these patients tended to be younger than 3 years. The five most frequent chief complaints resulting in an ED referral were fever, vomiting, lower abdominal pain, head injury, and viral infections.

**Table 6.** Most Frequent Diagnoses of Emergency Department Referrals

DIAGNOSIS	VISITS	PERCENT VISITS (MALE), %	AGE (AVERAGE)
Fever, unspecified [R50.9]	39	48.7	4.3
Vomiting, unspecified [R11.10]	10	50	1.7
Lower abdominal pain, unspecified [R10.30]	9	55.6	8.7
Unspecified injury of head, initial encounter [S09.90XA]	7	28.6	2
Viral infection, unspecified [B34.9]	7	42.9	4.6
Diarrhea, unspecified [R19.7]	6	50	8.7
Unspecified abdominal pain [R10.9]	6	100	7.2
Laceration without foreign body of lip, initial encounter [S01.511A]	5	80	5.6
Acute respiratory distress [R06.03]	3	33.3	0.3
Allergic urticaria [L50.0]	3	33.3	2

### COVID-19 TESTING

In addition to in-person care, many patients were referred to our urgent care offices for COVID-19 testing only. These visits did not include a second examination by an in-person provider. After a telemedicine visit, 14,659 patients were referred to our urgent care offices for COVID-19 polymerase chain reaction (PCR) testing, another 2,740 patients were referred for COVID-19 antibody testing, and 290 were referred for COVID-19 antigen testing. One hundred seventy-five patients were referred for both PCR and antibody testing.

## Discussion

As the COVID-19 pandemic created lockdowns and stay-at-home orders, our group of pediatric urgent care offices sought to continue providing acute care to children in our communities via a new telemedicine platform. In the first 12 months of launching our telemedicine platform, we saw more than 100,000 visits. For this study, we limited our scope to the period of 4 months where the platform was widely available in each state in which we had a physical presence. In that time, we saw 30,000 children from 0 through 18 years, with 6,214 children evaluated by a pediatric emergency physician and the remaining visits evaluated by providers experienced in pediatric urgent care.

Schinasi et al. showed that diagnostic accuracy is similar between virtual and in-person ED intake,^8^ which supports that our immediate-care model of telemedicine provides precise and correct care. In more than 90% of cases, we provided complete care within the telemedicine platform, with 9.2% (2,768) needing same-day urgent care and only 0.7% (219) referred to an ED. Children referred to the ED tended were younger and tended to present later in the evening, when both our telemedicine and in-person care was closing. Wait times on the platform were very low (most seen within 15 min), and chart completion time was also rapid (median chart completion was 16.3 min); thus, patients could receive care and view their electronic medical record quickly.

In studying transfer to urgent care, one limitation of our study is that we could only account for patients who presented to our own urgent care offices for in-person care. It is possible that others may have sought care elsewhere, and our estimates for patients who require same-day urgent care may be lower than the actual number of families who took their children in. Ideally, we would analyze patient satisfaction by surveys to determine if using our own institution for same-day in-person care is an accurate representation, however, only 3% of families completed the surveys that were sent after all telemedicine visits.

Our telemedicine platform not only provided rapid care to families, with a very short wait time, but given that chart completion had a median time of 16.3 min, it was also a rapid process for providers.

Our antibiotic prescription rate was 4.3%, which suggests that even in a time when COVID-19 made it impossible for many families to have in-person examinations, providers still adhered to being antibiotic stewards. Compared with a study of children seeking telemedicine care in 2019, where Ray et al. found that 52% of 4,604 telemedicine visits were prescribed oral antibiotics, our rates were significantly lower.^18^ In our study, oral antibiotics were most often for a skin infection, which can be easily assessed by video examination, as opposed to an illness such as otitis media, which cannot be assessed in a simple video examination.

In addition, we found higher rates of antibiotic prescribing in the beginning of the pandemic, which correlates with state shutdowns and fewer in-person health care options available. As shutdowns eased, conditions that could potentially require antibiotics were likely being referred to in-person care for complete physical examinations, which is reflected in a decrease in antibiotic prescribing.

Unlike existing telemedicine options, our platform specifically employs pediatric-trained providers who are also providers in our in-person pediatric urgent care where they adhere to nationally recognized standards for pediatric care. This may partly explain why our antibiotic prescribing rate was admirably low and suggests that the best telemedicine care for children should come from pediatricians and pediatric advanced practice providers.

Telemedicine has the potential to improve access to high-quality care to low-income and rural populations; limitations of this study include a patient population that was primarily the same patient population as our in-person offices. E-mails about this new service were sent to all prior patients, social media accounts were updated with the telemedicine option, and the main website was similarly updated. Nevertheless, despite the digital outreach and updates, we still mostly saw patients who were in close physical proximity to our urgent care offices. While ∼37% of the nation's children have public insurance, only 20.9% of our population had public insurance.

Our population was skewed to higher income groups because we did not have the ability to mass market to the broader community, and our 4-month study time period did not allow enough time for knowledge of the technology to spread naturally throughout our communities.

In addition, accessing telemedicine requires a fair amount of internet savvy, and since telemedicine is only available by application on a smart phone or on a personal computer with a webcam, we may have unintentionally excluded families without stable Wi-Fi and those who lacked the devices to run these programs. While our website is available in Spanish and we frequently see patients in office who speak Spanish and many other languages, we noted that we had a small percentage of foreign language speakers for our telemedicine visits.

An additional limitation of the study is that this occurred during a unique and evolving period of COVID-19 state-wide shutdowns and may not reflect standards of care outside this pandemic time. In our study period, many pediatricians near our physical offices were fully closed or were unable to accommodate sick visits. It is critical to note that over half of the care we provided was related to COVID-19; these were families looking for testing options for their children, or worried about potential COVID-19 exposures or symptoms. In many of our locations, PM Pediatrics was the only source of pediatric COVID-19 testing and care.

## Conclusions

Children can be well served by telemedicine with complete medical care while maintaining low antibiotic prescribing when pediatric trained providers are available. Many families will likely continue using telemedicine applications, even after the pandemic has passed, and thus, it is essential to define best practices in telehealth for the future. This study shows that telemedicine was transformative in the care of children during the COVID-19 pandemic, with the audiovisual examination being robust for the vast majority of chief complaints. In seeing 30,000 children in less than 4 months, we provided an essential public health service in the communities we serve.
